# A Comprehensive Evaluation of a Coumarin Derivative and Its Corresponding Palladium Complex as Potential Therapeutic Agents in the Treatment of Gynecological Cancers: Synthesis, Characterization, and Cytotoxicity

**DOI:** 10.3390/pharmaceutics16111437

**Published:** 2024-11-11

**Authors:** Mirela Jevtić, Marijana Stanojević Pirković, Teodora Komazec, Marija Mojić, Sanja Mijatović, Danijela Maksimović-Ivanić, Dušan Dimić, Zoran Marković, Dušica Simijonović, Dejan Milenković, Edina Avdović

**Affiliations:** 1 Department of Gynecology and Obstetrics General Hospital Uzice, Miloša Obrenovića 17, 31000 Užice, Serbia; jevticmirela@gmail.com; 2 Department of Medical Biochemistry, Faculty of Medical Sciences, University of Kragujevac, Svetozara Markovića 69, 34000 Kragujevac, Serbia; 3 Department of Immunology, Institute for Biological Research “Siniša Stanković”—National Institute of the Republic of Serbia, University of Belgrade, Bulevar Despota Stefana 142, 11108 Belgrade, Serbia; teodora.komazec@ibiss.bg.ac.rs (T.K.); marija.mojic@ibiss.bg.ac.rs (M.M.); sanjamama@ibiss.bg.ac.rs (S.M.); nelamax@ibiss.bg.ac.rs (D.M.-I.); 4 Faculty of Physical Chemistry, University of Belgrade, Studentski trg 12-16, 11000 Belgrade, Serbia; ddimic@ffh.bg.ac.rs; 5 Department of Natural Science and Mathematics, State University of Novi Pazar, Vuka Karadžića bb, 36300 Novi Pazar, Serbia; zmarkovic@uni.kg.ac.rs; 6 Department of Engineering and Natural Sciences, University of Applied Sciences Merseburg, Eberhard Leibnitz-Str. 2, 06217 Merseburg, Germany; 7 Department of Science, Institute for Information Technologies, University of Kragujevac, Jovana Cvijića bb, 34000 Kragujevac, Serbia; dusicachem@kg.ac.rs (D.S.); dejanm@uni.kg.ac.rs (D.M.)

**Keywords:** coumarin derivative, palladium(II) complex, DFT, HSA, cytotoxicity

## Abstract

**Background:** The aim of this research is the synthesis and characterization of coumarin-palladium complex and the investigation of the cytotoxicity of both the ligand and the complex. **Methods:** The palladium( II) complex (**CC**) was obtained in the reaction between (*E*)-3-(1-((4-hydroxy-3-methoxyphenyl)amino)ethylidene)-2,4-dioxochroman-7-yl-acetate (**CL**) and potassium-tetrachloropalladate(II) and characterized using IR and NMR spectra, experimentally and theoretically. Cytotoxicity of **CL** and **CC** were determined for human cervical carcinoma HeLa, ovarian cancer A2780, hormone dependent breast cancer MCF7, and colorectal cancer HCT116 lines. The interaction of investigated compounds with HSA was followed by spectrofluorimetric method. The binding mechanism in the active pocket was assessed via molecular docking simulations. **Results:** A low mean absolute error between experimental and theoretical data proved that the optimized structure corresponded to the experimental one. Both compounds showed a satisfactory selectivity index towards neoplastic cells. The binding affinity of tested compounds to the HSA were confirmed. The molecular docking showed a much lower change in the Gibbs free energy of binding for **CC** compared to **CL**. **Conclusions:** The obtained results revealed that **CL** and **CC** exhibit significant effects on several cancer cell lines and good binding properties to HSA, while molecular docking discovered that **CC** has the most pronounced activity against alpha-fetoprotein.

## 1. Introduction

Cancer is a complex and oxidative stress-related disease and one of the most prominent causes of death worldwide. The success of cisplatin complexes, although marked by side effects such as myelosuppression, peripheral neuropathy, nephrotoxicity, and fatigue, has led to using metal-containing compounds in medicine [1,2]. Palladium complexes also show similar chemical and biological behaviors to platinum complexes [3,4]. Many of these complexes have been described in the literature with promising anti-cancer activity; in some cases, they were more potent than their platinum analogs [5].

Coumarin (2*H*-1-benzopyran-2-one) and its metabolites are an important class of secondary metabolites isolated from the fruits, roots, flowers, and leaves of higher plants belonging to the *Umbelliferae*, *Oleaceae*, *Clusiaceae*, and *Rutaceae* families [6,7,8,9]. These compounds act as hormones and signaling molecules and function in the defense response to herbivores and microorganisms. Different coumarin compounds showed antibacterial, anticoagulant, antioxidant, antifungal, and cytotoxic properties [10,11]. Along with natural coumarin derivatives, their synthetic analogs have been a point of interest in modern synthetic chemistry [12,13]. Synthetic anticoagulant drugs were obtained from synthetic coumarins [14]. These analogs also inhibit monoamine oxidase, acetylcholinesterase, and butyrylcholinesterase, making them promising therapeutics for neurodegenerative diseases [15]. Other biological activities of synthetic coumarins include anti-HIV, antimicrobial, anti-inflammatory, and antitumor potency [16,17,18,19,20,21]. The antioxidant activity and reactivity of coumarin derivatives towards free radicals were extensively examined in references [22,23,24]. In technical applications, coumarins can be used as molecular photonic devices, dyes in light-emitting materials, and optical brighteners [25,26,27]. Coumarin–palladium(II) complexes were found to be active in different human cancer cell lines [28,29].

The synthesis and structural characterization of (*E*)-3-(1-((4-hydroxy-3-methoxyphenyl)amino)ethylidene)-2,4-dioxochroman-7-yl acetate ligand (**CL**) was previously described in [12]. The mechanisms of antiradical activity towards biologically important hydroxyl radicals were assessed using EPR and theoretical chemistry methods, and the importance of acid/base equilibrium species was outlined. Similar coumarin derivatives, namely 3-acetyl-4-hydroxycumarin, 3-(1-((3,4-dihydroxyphenethyl)-amino)ethylidene)-chroman-2,4-dione, and 3-(1-((2-hydroxyphenyl)amino)ethylidene) chroman-2,4-dione, also showed significant cytotoxic activity towards different cell lines [13,30,31].

The aim of this manuscript is to present structural and spectroscopic analyses of the newly obtained palladium(II) complex (**CC**) with **CL**. The theoretical ^1^H and ^13^C NMR spectra, along with vibrational spectra, were obtained upon optimization at the B3LYP-D3BJ/6-311+G(d,p) level of theory and compared to the experimental spectra to verify the predicted structure. The cytotoxicities of **CC** and **CL** were determined towards human cervical carcinoma HeLa, ovarian cancer A2780, hormone-dependent breast cancer MCF7, and colorectal cancer HCT116 lines. The mechanism of cell death was also determined. The HSA binding affinity of the compounds was examined via spectrofluorimetric titration, and the binding mechanism was elucidated using molecular docking simulations.

## 2. Materials and Methods

### 2.1. Chemicals

The chemicals 4,7 dihydroxycoumarin, 5-amino-2-methoxyphenol, phosphorus oxychloride DMSO-*d_6_*, chloroform-*d*, potassium tetrachloropalladate salt, ethanol, methanol, human serum albumin (HSA), phosphate-buffered saline (PBS), diacetate succinimidyl ester (CFSE), dihydroethidium (DHE), propidium iodide (PI), ribonuclease A (RNaseA), and crystal violet (CV) were purchased from Sigma-Aldrich (St. Louis, MO, USA). The following reagents were used for cell propagation, treatment, and flow cytometry: RPMI 1640 culture medium (with L-glutamine and 25 mM HEPES), RPMI 1640 culture medium without phenol red, DMEM high-glucose culture medium, fetal bovine serum (FBS), and trypsin/ethylenediaminetetraacetic acid (EDTA); these were obtained from Capricorn Scientific GmbH (Ebsdorfergrund, Germany). Dimethyl sulfoxide (DMSO) and paraformaldehyde (PFA) were obtained from Serva Electrophoresis GmbH (Heidelberg, Germany). 3-(4,5-Dimethythiazol-2-yl)-2,5-diphenyltetrazolium bromide (MTT), and dihydrorhodamine 123 (DHR) were purchased from Thermo Fisher Scientific (Waltham, MA, USA). 4-Amino-5-methylamino-2′,7′-difluorofluorescein diacetate (DAF-FM) was purchased from Enzo Life Sciences (Farmingdale, NY, USA). The penicillin/streptomycin solution was purchased from Biological Industries (Cromwell, CT, USA). Annexin V-FITC (AnnV) was acquired from BD Biosciences Pharmingen (San Diego, CA, USA). ApoStat was obtained from R&D Systems (Minneapolis, MN, USA). Dimethyl sulfoxide (DMSO) for cells was purchased from Santa Cruz Biotechnology (Dallas, TX, USA).

The **CL** and **CC** stocks (50 mM) were prepared in DMSO and stored at −20 °C before use, while the cisplatin stock (10 mM) was prepared in DMF and always used fresh.

### 2.2. Cells

HeLa (human cervical adenocarcinoma cells), A2780 (human ovarian carcinoma cells), MCF7 (human breast adenocarcinoma cells), and HCT116 (human colorectal carcinoma cells) were purchased from American Type Culture Collection (ATCC, Rockville, MD, USA). HeLa, MCF-7, and HCT116 cells were cultivated in HEPES-buffered RPMI-1640, while A2780 cells were cultivated in DMEM. The media were supplemented with 10% heat-inactivated FBS and antibiotics (100 units/mL penicillin and 100 μg/mL streptomycin). The cells were grown at 37 °C in a humidified atmosphere with 5% CO_2_. For the viability assays, HeLa (3 × 10^3^/well), A2780 (8 × 10^3^/well), MCF7 (7 × 10^3^/well), and HCT116 (6 × 10^3^/well) cells were seeded in adherent flat-bottom 96-well plates. For the flow cytometric analyses, HeLa cells were seeded at 8 × 10^4^ in 6-well plates.

Peritoneal exudate cells (PECs) were isolated from the peritonea of 8–12-week-old C57BL/6 mice via a cavity rinse with ice-cold PBS. Cells were seeded in HEPES-buffered RPMI-1640 medium supplemented with 5% heat-inactivated FBS and antibiotics. The animals were from the animal facility of the Institute for Biological Research “Siniša Stanković” (IBISS) National Institute of the Republic of Serbia (Belgrade, Serbia). Animal handling was conducted and protocols were followed by the local Institutional Animal Care and Use Committee (IACUC) in accordance with local guidelines as well as European Community directives (EEC Directive of 1986; 86/609/EEC). The ethical approval number is 323-07-02147/2023-05.

### 2.3. Instrumentation

The elemental microanalysis of C, H, and N was performed on the Elemental analyzer ECS 8020, CHNS–O (Valencia, CA, USA). The measurement was conducted in triplicate at the Institute for Chemistry, University of Kragujevac. For IR spectral measurements, a Perkin–Elmer Spectrum spectrophotometer (Shelton, WA, USA) as employed using the KBr pellet technique (range of wavenumbers 4000–400 cm^−1^). ^1^H and ^13^C NMR spectral analyses were carried out using a Varian Gemini-2000 spectrometer (^1^H NMR at 200 MHz; ^13^C NMR at 50 MHz) at 298 K in DMSO-d_6_ with tetramethylsilane (TMS) as an internal standard. A fluorescence spectrophotometer, Shimadzu, RF-6000 (Tokyo, Japan), was used for the fluorescence experiments. This instrument was equipped with a xenon lamp source, a temperature controller, and a 1.0 cm cell.

### 2.4. Chemical Studies

#### General Procedure for the Synthesis of Coumarin Complex **CC**

A previously synthesized and fully characterized ligand [12] was used to synthesize a new palladium complex. An aqueous potassium tetrachloropalladate(II) and a methanol solution of bidentate coumarin ligand were utilized to form a coumarin–palladium(II) complex, **CC**. For six hours at room temperature, solutions of the ligand and palladium(II) salts were continuously stirred on a magnetic stirrer [32,33]. A moderately good yield of the novel palladium(II) complex was produced following precipitation, filtering, and air drying. After several attempts, the crystallization of the obtained complex was unsuccessful, and its X-ray crystallographic structure could not be determined. Spectral characterization of **CL** and **CC** is given in the manuscript, while their corresponding spectra are shown in the Supplementary Materials.

(*E*)-3-(1-((4-hydroxy-3-methoxyphenyl)amino)ethylidene)-2,4-dioxochroman-7-yl acetate (**CL**): yield, 0.389 g (77.42%). **^1^H NMR** (200 MHz, DMSO-d6) δ ppm: 2.31 (3H, s, C4′–H), 2.60 (3H, s, C2′–H), 3.36 (3H, s, OCH_3_), 6.86 (1H, m, C5″–H), 7.03 (2H, m, C2″–H, C6″–H), 7.13 (2H, m, C6–H, C8–H), 8.01 (1H, d, H-5, H-6 = 8.5 Hz, C5–H), 9.49 (1H, s, OH), 15.25 (1H, s, NH). **^13^C NMR** (50 MHz, DMSO-*d6*) δ ppm: 20.62 (C2′), 21.06 (C4′), 56.05 (OCH3), 96.70 (C3), 109.87 (C2″), 110.10 (C8), 115.62 (C10), 117.71 (C5″), 118.02 (C6″), 118.07 (C6), 127.10 (C5), 127.14 (C1″), 146.21 (C4″), 148.21 (C3″), 153.83 (C7), 154.85 (C9), 161.48 (C2), 168.66 (C1′), 175.82 (C3′), 179.51 (C4). **IR (KBr)** ν cm^−1^: 3327 (O–H), 3064 (N–H), 1767, 1693 (C=O), 1622, 1572, 1512, 1464 (C=C), 1207 (C–O).

*Bis*-3-(1-((4-hydroxy-3-methoxyphenyl)amino)ethylidene)-2,4-dioxochroman-7-yl acetate palladium(II) complex (**CC**): yellow powder; yield, 0.062 g (46%). Anal. calc. for C_40_H_32_O_14_N_2_Pd (Mr = 871.12) %: C, 55.15; H, 3.70; N, 3.22. Found: C, 51.55; H, 3.75; N, 3.60. **^1^H NMR** (200 MHz, DMSO-d6) δ ppm: 2.18 (s, 6H, C–4′), 2.31 (s, 6H, C–2′), 3.75 (s, 6H, OCH_3_), 6.73–6.95 (m, Ar, 4H), 7.04–6.95 (m, Ar, 1H), 7.10 (d, *J* = 2.1 Hz, Ar, 1H), 9.19 (s, 1H, OH). **^13^C NMR** (50 MHz, DMSO-*d6*) δ ppm: 20.54 (C–4′), 21.06 (C–2′), 55.97 (OCH_3_), 96.76 (C–3), 109.85 (C–10), 112.52 (C–8), 112.78 (C–5″), 116.34 (C-6″) 117.68 (C–2″), 118.05 (C–5), 127.11 (C–6), 128.63 (C–1″), 147.21 (C–4″), 147.71 (C–3″), 153.82 (C–9), 154.85 (C–7), 161.44 (C–3′), 168.65 (C–2), 175.67 (C–1′), 179.54 (C–4). **IR (KBr)** ν cm^−1^: 3263 (O–H), 3057 (N–H), 1750, 1683 (C=O), 1572, 1434 (C=C), 1257, 1207 (C–O), 564 (Pd–O); 422 (Pd–N).

### 2.5. DFT Studies

The geometry optimizations of the ligand and complex were carried out using density functional theory (DFT) employing the functional B3LYP with empirical dispersion corrections D3BJ (with Becke and Johnson damping) [34,35]. For the non-metallic atoms, a 6–311+G(d,p) basis set was used, while for Pd, the def2-TZVPD basis set was chosen [36], as implemented in Gaussian 09 software [37]. The structures were optimized at 298 K without any geometrical restrictions. The equilibrium geometries had no imaginary vibrations. The complex **CC** can exist in two conformations, *cis* and *trans*. The relative abundance of the conformers was determined based on their energies through the Boltzmann distribution formula used for this purpose:

(1) $$\frac{N_{CIS}}{N_{trans}}=e^{-\left(G_{cis}-G_{trans}\right)}/RT$$

where N_cis_, N_trans_, G_cis_, G_trans_, k, and T represent the number of particles in each state, the Gibbs free energy of both conformations, the Boltzmann constant, and the temperature, respectively [38].

### 2.6. Biological Studies

#### 2.6.1. Assessment of Cellular Viability: MTT and CV Assays

The cells were treated with a range of CL, CC, and cisplatin concentrations for 72 h. At the end of the treatment, the cell viability was determined using MTT and crystal violet assays, as previously described [39]. PECs were seeded at a density of 1.8 × 10^5^ cells/well in 96-well plates, and two hours later, non-adherent cells were removed by washing the wells with PBS. The remaining adherent cells were treated the same way as the tumor cells. The PEC viability was estimated after 72 h of treatment using the CV assay only.

#### 2.6.2. Flow Cytometry

HeLa cells were treated with IC_50_ values of **CL** (17 μM), **CC** (20 μM), and cisplatin (0.5 μM) for 72 h. To analyze the distribution of cells in the different phases of the cell cycle using flow cytometry, cells were collected at the end of the treatment and processed as previously described [40]. To detect the presence of apoptotic cells, the treated HeLa cells were double-stained with Annexin V-FITC/PI in Annexin V-binding buffer according to the manufacturer’s instructions. For caspase activation, cells were incubated with FITC-labeled pan-caspase inhibitor ApoStat (0.5 μg/mL) for 30 min at 37 °C. The cells were then washed, resuspended in PBS, and analyzed using flow cytometry. The effect of the compounds on cell proliferation was analyzed via CFSE staining using flow cytometry in the same manner as previously described [40]. DAF-FM was used to determine the intracellular production of nitric oxide in the treated HeLa cells, DHE was used to detect intracellular superoxide and hydrogen peroxide, and DHR123 was used to detect the cumulative intracellular production of reactive oxygen species and nitrogen species. These assays were performed as described in previously published studies [39]. All flow cytometry data were obtained in a CytoFLEX cytometer (Beckman Coulter, Brea, CA, USA) and analyzed using FlowJo software v10 (Tree Star, Ashland, OR, USA).

#### 2.6.3. Statistical Analysis

Data were acquired from three independent experiments and analyzed using one-way analysis (ANOVA) with Bonferroni correction. A *p*-value less than 0.05 was considered statistically significant.

### 2.7. Fluorescence Spectroscopy Experiment

The fluorescence spectroscopy method was used to examine the possible binding ways of human serum albumin (HSA) with the investigated compounds. These experiments were carried out at three different temperatures (296, 303, and 310 K) with a fixed 2.0 µM HSA concentration, while the concentrations of ligands and complexes were serially increased in the range of 0–10 μM. The fluorescence of HSA was recorded at an excitation wavelength of 290 nm, while the recording range was set at 300–500 nm. The gradual decrease in the maximum emission intensity of the HSA with an increasing concentration of all investigated compounds was monitored at about 330 nm.

Fluorescence quenching parameters were determined using the Stern–Volmer equation (Equation (2)):

(2) $$\frac{I_{0}}{I}=1+k_{q}\tau_{0}\left[Q\right]=1+K_{SV}\left[Q\right].$$

The notations in the previous equation include *I*_0_ and *I* as HSA fluorescence intensities in the absence and presence of the quencher, while *K_SV_* and *k_q_* are the Stern–Volmer quenching constant and the quenching rate constant of HSA, *τ*_0_ is the fluorophore lifetime of HSA (in the absence of the quencher), and [*Q*] is the concentration of the quencher. The binding constant (*K_b_*) and the number of binding sites (*n*) were calculated using the Hill equation (Equation (3)) [41]:

(3) $$log\frac{I_{0}-I}{I}=logK_{b}+nlog\left[Q\right].$$

where *I*_0_ and *I* represent the HSA fluorescence intensities in the absence and presence of the quencher, and [*Q*] is the concentration of the quencher.

The thermodynamic parameters Δ*H*°, Δ*S*°, and Δ*G*° were calculated using the following equations:

(4) $$lnK_{b}=-\frac{∆H^{{}^{\circ}}}{RT}+\frac{∆S^{{}^{\circ}}}{R}$$

(5) $$∆G^{{}^{\circ}}=∆H^{{}^{\circ}}-T∆S^{{}^{\circ}}$$

### 2.8. Molecular Docking Simulations

Molecular docking simulations were performed to calculate the binding affinity of the ligand and its corresponding complex against the three different proteins. The receptor pockets and binding sites were determined using the AMDock program [42]. The crystal structures of the **HSA**, **AFP** (tumor marker of ovarian cancer), and **CEA** (tumor marker of breast and colorectal cancers) receptors (PDB: 7jwn) were downloaded from the RCSB Protein Data Bank in pdb format [43]. The structures were prepared for the docking simulations in The Discovery Studio 2020 [44], according to the paper by Milenković et al. 2020 [45]. The polar hydrogen atoms and Kollman united atom partial charges for all amino acids were added subsequently using the AutoDock Tools (ADT) graphical interface [46], as required in the Lamarckian Genetic Algorithm (LGA). The grid center with the dimensions 8.579 × 15.016 × 19.323 Å in the -x, -y, and -z directions of the protein target was employed to cover the protein binding site and allow the ligand to move freely. To examine the binding affinity of the investigated compounds, AutoDock 4.2 software was used.

## 3. Results and Discussion

### 3.1. Chemical Studies

**Scheme 1:**
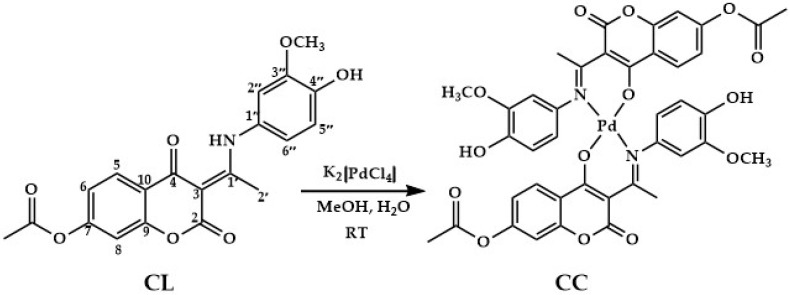
The general reaction for the synthesis of the complex **CC**.

Coumarin ligands have been shown to exhibit strong coordination with transition metal ions, making them valuable tools in coordination chemistry. The synthesis and comprehensive characterization of bidentate coumarin ligand **CL** have previously been reported [12]. To obtain its corresponding palladium(II) complex **CC**, a methanol solution of bidentate coumarin ligand and an aqueous solution of potassium tetrachloropalladate(II) were used [32,33] (Scheme 1).

### 3.2. Spectroscopic and DFT Characterization

The structural properties of the examined ligand were established in prior publications using nuclear magnetic resonance (NMR) and infrared (IR) spectroscopy, elemental analysis, and density functional theory (DFT) methodologies. This study has yielded useful knowledge regarding the ligands’ chemical composition and molecular structure, which are crucial for comprehending their behavior in complexation reactions with transition metals. A new complex was synthesized to evaluate the biological activity of the coumarin derivative and its corresponding palladium(II) complex, and the cytotoxicity of both the ligand and complex was assessed. The obtained complex was analyzed using nuclear magnetic resonance, infrared spectroscopy, and DFT methods to confirm the assumed structure. The results showed that a stable complex with similar cytotoxicity as the ligand was obtained. Based on the crystallographic structures of similar complexes [33,47], it was determined that the bidentate ligands bind to the palladium(II) ion only through the oxygen atom of the carbonyl group at position 4 and the deprotonated nitrogen atom of the secondary amino group, creating a stable six-membered ring. A great similarity with the previously synthesized complexes was observed in the obtained NMR spectra. Namely, in the proton spectra of the complex, as in the previous cases, the absence of a singlet was observed at around 15 ppm. These signals originate from secondary amino groups in the ligands. This fact indicates that the coordination of the ligand toward the palladium(II) ion is similar to the previous results. In addition, the presence of two singlets of the methyl groups at about 2 ppm, and characteristic peaks in the aromatic region further confirms the structure of the complex (Figure S1). Differences in the chemical shifts in the ^13^C NMR spectra provided significant information about ligand coordination to the Pd(II) ion. The differences in the chemical shifts of the carbon atom signals in the NMR spectra after coordination (Δδ_coord._, complexation coefficient) were calculated according to the relation Δδ_coord._ = δ_complex_ − δ_ligand_ [28] (Avdović, 2023). The most significant changes were observed in the chemical shifts of the signals of the three carbon atoms: C1′, C1″, and C2′ (Δδ_coord._ = −7.01 ppm, −1.49 ppm, and −0.44 ppm, respectively) (Figure S2). Changes in the chemical shifts in the ^1^H and ^13^C NMR spectra concerning the ligand are a consequence of the change in the electronic environment of these atoms, which occurs due to the formation of new coordinate covalent bonds in the complex. The different electronic environment within the complex suggests completely different properties than the ligand. The IR spectra also showed bands corresponding to vibrations of the OH group within the range of 3263–3057 cm^−1^ and stretching vibrations of the lactone carbonyl groups within the range of 1750–1683 cm^−1^. The band assigned to NH vibrations was not observed in the IR spectrum of the complex, unlike the spectra of the ligand. The bands observed at 1572 and 1434 cm^−1^ are attributed to the stretching vibrations of C=N and C=C bonds. Additionally, 564 and 422 cm^−1^ bands correspond to the stretching vibrations of Pd–O and Pd–N bonds, respectively. Overall, the NMR and IR spectra of the complex agree with the results of previous studies and confirm the bidentate binding of the ligand to the Pd(II) ion.

#### 3.2.1. DFT Characterization

**Figure 1:**
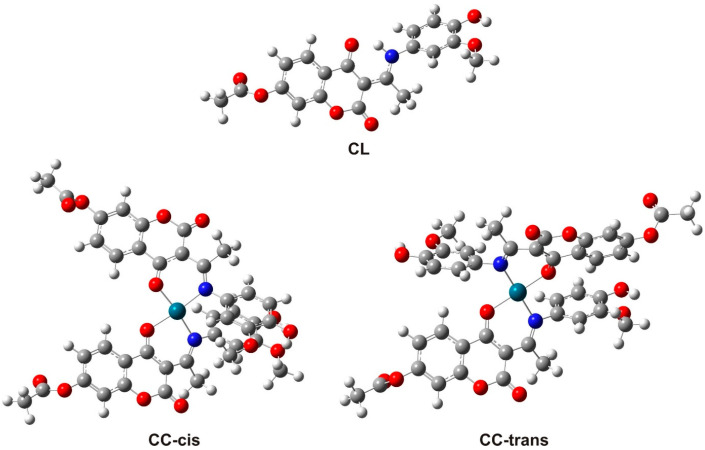
The optimized structures of the investigated *cis*–**CC** and *trans*–**CC**.

Ligand **CL** and *cis* and *trans* isomers of complex **CC** were optimized using the DFT theoretical model in DFT methodology (Table S2). Their geometries are shown in Figure 1. The results showed that the *trans* isomer is more stable as the Gibbs free energy value is 15.9 kJ mol^−1^ lower than that of the *cis* isomer. The dominant presence of the ***trans*–CC** isomer is confirmed by the Boltzmann distribution value of 98.97%. The difference in the stability of these two isomers is due to steric hindrance and electronic repulsion between the aromatic rings.

The optimized structures of investigated compounds are presented in Figure 1. The calculated bond lengths and angles are listed in Table S2. Using the τ_4_ formula, which quantifies the continuum of four-coordinate geometries between ideal tetrahedral (*T*_d_) and ideal square-planar geometries (*D*_4*h*_) [48], we found that **CC–*cis*** is stabilized with a slight increase in the distortion of the square planar geometry (τ_4_ = 0.11), while **CC–*trans*** is stabilized with the least distortion from square planarity (τ_4_ = 0.05) due to coordination with the two N and the two O atoms in trans configuration and the diagonal angles, _(C2′-)_N-Pd-N_(–C2″)_ (176.3) and _(C4–)_O-Pd-O_(–C4′)_ (176.1), around the palladium atom. The Pd-O and Pd-N bond lengths are 1.991 Å (_(C4–)_O-Pd), 2.006 Å (_(C4′–)_O-Pd), 2.040 Å (_(C2′–)_N-Pd), and 2.050 Å (_(C2″–)_N-Pd) (Table S3).

#### 3.2.2. Vibrational Spectra Analysis

**Figure 2:**
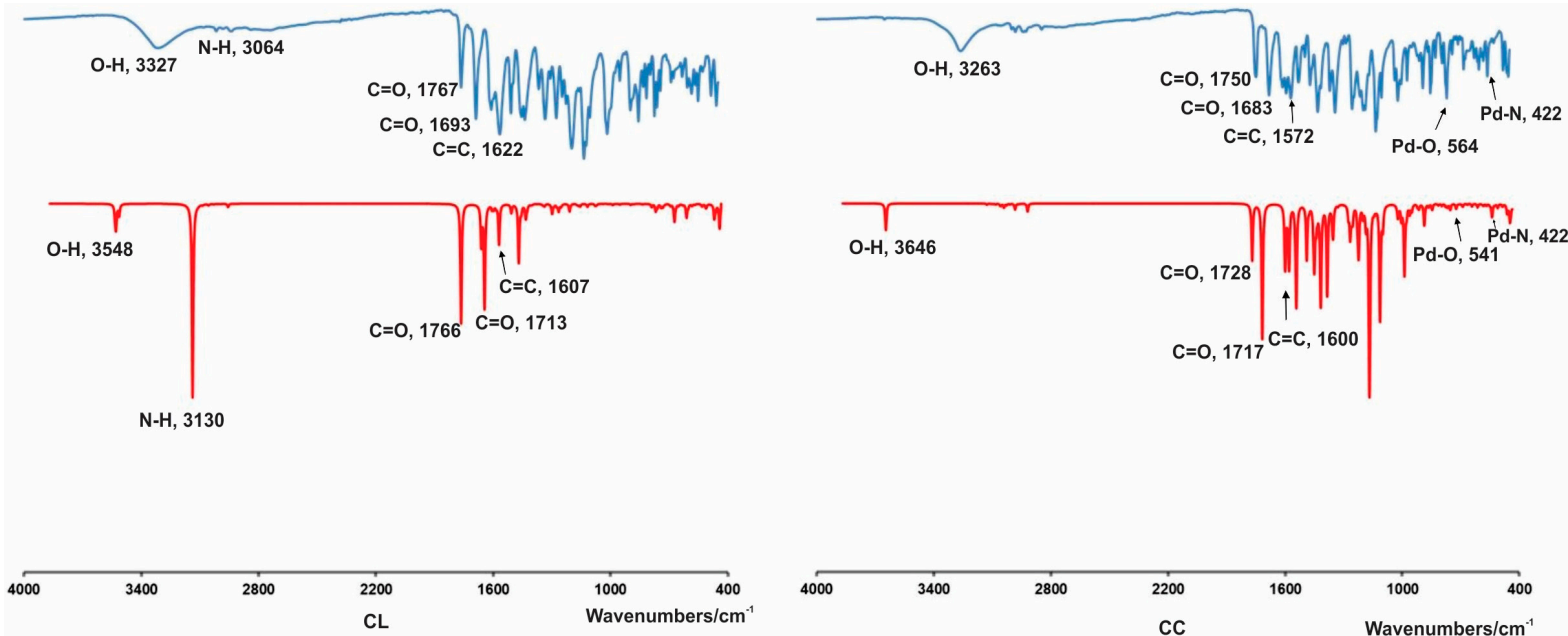
IR spectra of the compounds under investigation: the experimental (blue line) and theoretical (red line).

Vibrational spectroscopy is an important structural characterization method [49,50,51]. The most prominent bands in the **CL** and **CC** spectra originate from the characteristic vibrations of the benzene and pyrone rings and the carbonyl groups. Figure 2 displays experimental and theoretical spectra of both the pure ligand and complex. These spectra allowed for structural characterization of the mentioned compounds. A thorough comparison is performed only for **CL** because, due to the symmetry of the complex, no significant differences are expected in vibrational modes except for the groups involved in complexation (Pd–O and Pd–N vibrations). After comparing the experimental and theoretical wavenumbers, it was concluded that the computed wavenumbers were generally higher than the observed data. Therefore, the calculated wavenumbers were scaled with a scaling factor of 0.9670, as shown in reference [51]. After scaling, there was a decrease in the discrepancy between the experimental and theoretical wavenumber values (correlation coefficient of 0.999 and an average absolute error of about 3 cm^−1^). B3LYP-D3BJ/6-311+G(d,p) is, therefore, a suitable level of theory for the examination of the structure and intramolecular interactions of the ligand and complex in this contribution.

The experimental O–H stretching vibrations are observed at 3327 cm^−1^ in **CL** and 3263 cm^−1^ in **CC** compared to the predicted values at 3548 and 3646 cm^−1^. The polar O–H bond usually shows strong and broad absorption bands from 3200 to 3650 cm^−1^ (Figure 2) compared to the predicted values at 3548 and 3646 cm^−1^, whereas the predicted spectrum displays one sharp band. The CL’s N–H stretching mode is positioned at 3130 and 3064 cm^−1^ in the theoretical and experimental spectra, respectively. One of the reasons for the difference between these values is the formation of intramolecular hydrogen bonding interactions. Due to the complexation of the central metal ion, in the low-frequency region (below 1000 cm^−1^) of the infrared spectrum of **CC**, new bands appear at 564 cm^−1^ (Pd–O) and 422 cm^−1^ (Pd–N) (Figure 2). These bands can be found in the simulated spectrum at 541 and 432 cm^−1^. The coordination position is determined through the absence of an N–H stretching vibration in the spectrum of **CC** (Figure 2). The carbonyl group stretching vibration band is expected to be a medium or strong band between 1850 and 1550 cm^−1^ based on the physical state, electronic effects of neighboring substituents, conjugation, and hydrogen bonding [52]. Delocalization of π electrons additionally stabilizes the structures of the ligand and complex. The double-bond character of the C=O group is reduced for the mentioned reasons, resulting in an absorption at lower wavenumbers. The FTIR spectrum of **CL** shows two very strong bands at 1767 and 1693 cm^−1^ that are attributed to C=O stretching vibrations, which compares well with the calculated values of 1766 and 1713 cm^−1^. The C=O stretching vibration in **CC** is shifted to lower wavenumbers (observed at 1750 and 1683 cm^−1^ and calculated at 1718 and 1717 cm^−1^) due to the weakening of the bond and coordination with Pd, which is an additional proof of the complexation mode. In the experimental spectra of the ligand and complex, the C=C stretching vibrations appear at 1622, 1572, 1512, and 1464 cm^−1^ (**CL**) and 1572 and 1434 cm^−1^ (**CC**). The DFT/B3LYP-D3BJ method provides the same vibrations between 1607 and 1475 cm^−1^ in **CL** and between 1600 and 1477 cm^−1^ in **CC**. The simultaneous activation of the C=C stretching mode of the benzene ring in the examined spectrum provides evidence for the charge transfer interaction between groups through the π electron system.

#### 3.2.3. ^1^H and ^13^C NMR Spectrum Analysis

The investigated compounds’ ^1^H and ^13^C NMR spectra were recorded at 200 and 50 MHz using DMSO-*d_6_* as the solvent. Simulated NMR spectra were used to validate the proposed geometries of the obtained compounds. For this purpose, the optimized geometry of **CL** at the B3LYP-D3BJ/6-311+G(d,p) level of theory was used. This theoretical model was chosen because it agreed well with experimental IR spectra. The NMR simulation was conducted using the Gauge-Independent Atomic Orbital (GIAO) approach. The structure of **CL** was reoptimized in DMSO before conducting NMR simulations [53]. To assess the degree of similarity between the experimental and simulated spectra, we computed the correlation coefficient (R) and mean absolute error (MAE).

Based on the data provided in Table 1, the correlation coefficients and mean absolute errors suggest that the simulated spectra exhibit a very strong correlation with the experimental data. The protons bonded to aromatic and aliphatic carbon atoms showed excellent correlation. Similar conclusions can be reached from the study of the ¹³C NMR data. This demonstrates that the chosen DFT approach is suitable for modeling NMR spectra, thereby verifying the structure of the examined molecules.

### 3.3. Biological Studies

A panel of cells representing female reproductive organs or tissues functionally or spatially connected to them—human cervical carcinoma HeLa, ovarian cancer A2780, hormone-dependent breast cancer MCF7, and colorectal cancer HCT116—were exposed to newly designed **CL** and **CC** for 72h. After the indicated time point, their viability was assessed using two assays based on the different parameters used to quantify viable cells: the MTT and CV test.

As presented in Figure S3 and Table S1, the compounds dose-dependently decreased the number of viable cells. At the same time, IC_50_ values calculated from the data obtained using the MTT (3-(4,5-Dimethylthiazol-2-yl)-2,5-Diphenyltetrazolium Bromide) and CV (crystal violet) assays revealed differences in the sensitivities of the tested cell lines, with the most sensitive values found on HeLa cells and the least sensitive cells for colon adenocarcinoma HCT 116.

The cytotoxicity evaluation performed on primary mouse peritoneal exudate cells (Figure S4) revealed a significantly lower sensitivity to the same dose range and, thus, a satisfactory selectivity index (SI) varying from 1.6 to 5.5 towards neoplastic cells. Besides cisplatin exerting stronger activity against the same cell lines under comparable experimental conditions in vitro, the high toxicity towards primary cells and tissues in vivo drastically limited its application [54,55]. Accordingly, the coumarin derivative and its metal-based counterpart were deemed worthy of further investigation.

**Figure 3:**
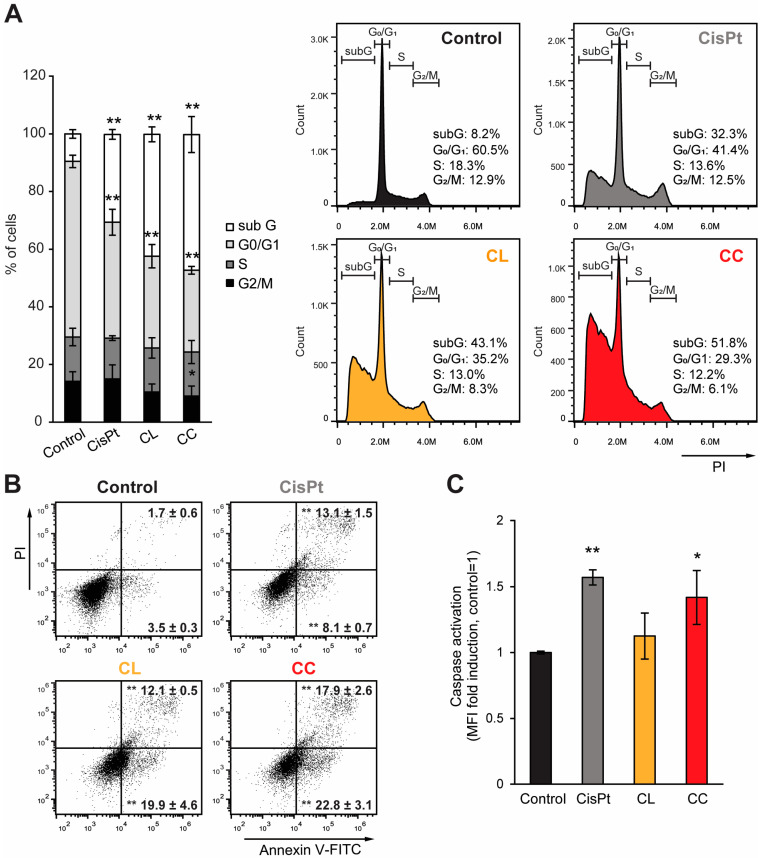
Effects of **CL** and **CC** on cell cycle distribution and apoptotic process. Human cervical adenocarcinoma cells (HeLa) were treated with an IC_50_ dose of **CL**, **CC**, or cisplatin (CisPt), whereas the controls were left untreated. After 72 h, the cells were analyzed using flow cytometry. (**A**) Cell cycle distribution presented as the mean ± SD of at least three independent experiments (**left**). Representative flow cytometry histograms from one of three independent experiments are shown (**right**). (**B**) Representative flow cytometry dot plots from one of three independent Annexin V-FITC/PI double staining experiments. The percentage of early (Ann + PI−) and late (Ann+PI+) apoptotic cells is shown as the mean ± SD from 3 repeated experiments. (**C**) Caspase activation analysis shown as the mean fluorescent intensities (MFI) of ApoStat normalized to the control. Presented are the mean ± SD of at least three independent experiments. *—*p* < 0.05 and **—*p* < 0.01 in comparison to the control, one-way ANOVA with Bonferroni correction.

To define the basic mechanism behind the antitumor activities observed in vitro, the influence of the tested compounds on cell cycle distribution, the presence of apoptotic and necrotic cell death, and overall caspase activation were investigated. As can be seen in Figure 3A, a strong DNA fragmentation, visualized by the accumulation of hypodiploid cells in the sub-compartment, was detected in the cultures exposed to all treatments, cisplatin, **CL**, and **CC**, but to a significantly greater extent with **CL** and especially **CC**. There was no substantial arrest in the S or G2/M phase of the cell cycle (Figure 3A). Concordantly, the CFSE staining used to measure cell division rate did not detect any increase in the undivided cell subpopulation compared to control untreated cells (Figure S5). Additionally, in agreement with the accumulation of hypodiploid cells in the SubG compartment, staining with AnnV together with PI as the counterstain, which allowed for differentiation between early and late apoptotic/necrotic cell death, showed an intense apoptotic process in the presence of the tested compounds, even more intense than that observed with cisplatin (Figure 3B). The apoptotic process was more or less accompanied by caspase activation in all treatments, suggesting that the intensity of apoptosis does not necessarily correlate with the intracellular level of caspase activation (Figure 3C).

**Figure 4:**
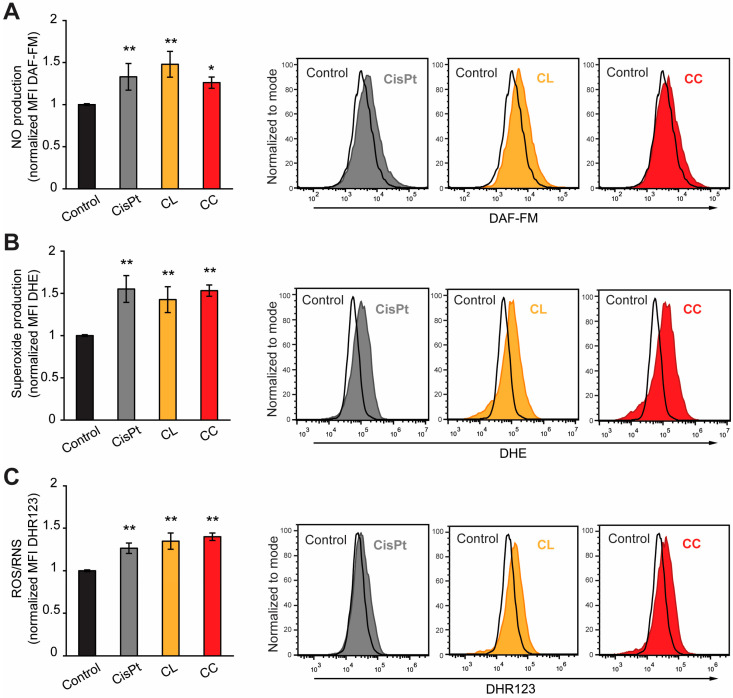
Effects of **CL** and **CC** on ROS/RNS production in HeLa cells. HeLa cells were exposed for 72 h to an IC_50_ dose of the tested drugs and subsequently analyzed using flow cytometry. (**A**) Intracellular NO production was detected using DAF-FM staining. (**B**) Superoxide anion radical production was detected with DHE staining. (**C**) DHR123 was used for the detection of reactive oxygen (ROS) and nitrogen (RNS) species. (**A**–**C**) The mean fluorescent intensities (MFI) normalized to the control are presented as the mean ± SD of at least three independent experiments (lefts). Representative flow cytometry histograms from one of three independent experiments are shown (right). *—*p* < 0.05 and **—*p* < 0.01 in comparison to the control, one-way ANOVA with Bonferroni correction.

Reactive oxygen species (ROS) function as regulators of crucial cellular processes, although they were previously considered by-products of aerobic metabolism [56]. This group of mediators has an important role in intracellular signal transmission and includes different oxygen-centered species (hydroxyl (HO^•^) and superoxide (O_2_^•^) radicals and hydrogen peroxide (H_2_O_2_)). Hydrogen peroxide is less reactive than most ROS but is able to reach every cell compartment and, acting as a second messenger, proceeds extracellular signals or controls of gene expression [57]. The redox status of malignant cells differs markedly from that of nontransformed equivalents, and the baseline production of free radicals is significantly higher compared to healthy cells. Thus, antioxidant intake can interfere with the metabolism and proliferation of cancer cells [58]. However, when the production of ROS overcomes the cell redox protection network, these molecules lead to cell damage and, subsequently, death. On the long list of chemotherapeutic agents, only a few act by inducing oxidative stress, and cisplatin is one of them [59]. Measurement of the intracellular production of nitric oxide and superoxide anion, hydrogen peroxide, and peroxynitrite revealed that newly synthesized therapeutics induce intracellular production of all mentioned reactive species similarly or even more efficiently than cisplatin (Figure 4). Accordingly, their potential to trigger apoptotic cell death may be closely related to the oxidative stress caused by increased NO, superoxide anion, and ROS/RNS production.

Altogether, experimental therapeutics based on 4-hydroxycoumarin derivatives affected tumor cell viability preferentially through oxidative stress induction and consequent caspase-dependent apoptotic cell death, but with significant selectivity obtained toward a malignant phenotype, providing them an advantage over cisplatin.

### 3.4. Spectrofluorimetric Investigation of HSA Binding Properties

To obtain information on the quenching, binding, and thermodynamic parameters for the interaction of the ligand and complex (**CL** and **CC**) towards human serum albumin (HSA), spectrofluorimetric investigations were carried out. All experiments were performed under physiological conditions in phosphate-buffered saline (PBS) and at different temperatures (296 K, 303 K, and 310 K).

#### 3.4.1. Analysis of Fluorescence Quenching

**Figure 5:**
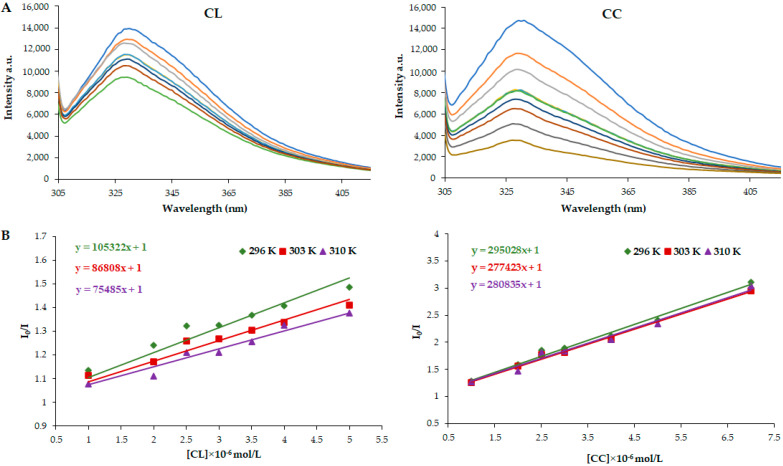
(**A**) Emission spectra of HSA in the presence of the corresponding compound (**CL** and **CC**). (**B**) The Stern–Volmer graph used to calculate the K*_SV_* and quenching constant k_q_ at different temperatures.

As indicated in Figure 5, a gradual decrease in the fluorescence intensity of the fluorophore was detected when the concentration of the quenchers increased. The fluorescence quenching phenomenon is caused by changes in the microenvironment of chromophores due to different molecular interactions of HSA with the investigated compounds. These interactions can be characterized by the complex formation between the fluorophore and quencher at the ground state via the static mechanism and the collision of the fluorophore and quencher in the excited state via the dynamic mechanism [41]. The relationship between the quenching constant and temperature can be used to distinguish quenching mechanisms. The quenching constant values are inversely correlated with temperatures in the static mechanism, while in dynamic quenching, K_SV_ values increase with a temperature rise [41]. In addition, a higher value of k_q_ than the maximum limit of 2 × 10^10^ M^−1^ s^−1^ indicates a binding-controlled static process with complex formation.

The values for K_SV_ and k_q_ were obtained using the Stern–Volmer quenching equation and are listed in Table 2. The good HSA binding propensities of both the ligand and complexes were proven by the obtained values. The lowering of the K_SV_ with an increase in temperature supported the static quenching process that led to ground-state complex formation in both cases. Also, the emission maximum wavelength was shifted towards lower values as the concentration of the compounds studied increased. This blue shift is caused by a change in the hydrophobicity of the environment of the binding region serum albumin.

#### 3.4.2. Binding and Thermodynamic Parameters

**Figure 6:**
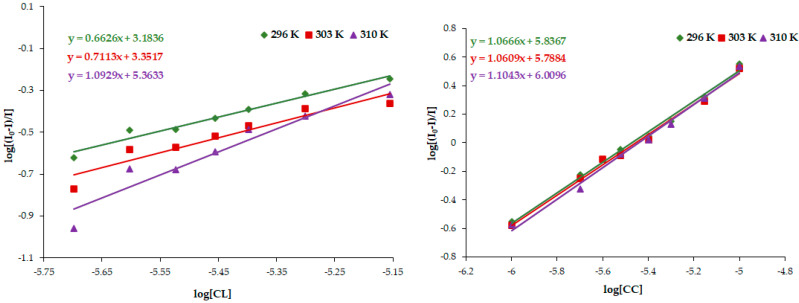
Hill plot for binding between compounds **CL** and **CC** with HSA at different temperatures.

The binding constants (K_b_) and binding stoichiometry (n) were calculated from Equation (2) (the double logarithmic plot of log[(I_0_ − I)/I] vs. log[Q]), as presented in Figure 6.

The binding constants and n values at three different temperatures (296, 303, and 310 K) were determined from the intercept and slope of these plots (Table 2). As evident from Table 2, the binding constants decreased with the increasing temperature of compound **CC**. Where the ligand is concerned, higher binding constants were also characteristic of the higher temperatures, indicating that protein can accommodate this ligand better at 310 K than at 296 K. The stoichiometries of both investigated binding systems are close to one, which indicates a 1:1 binding ratio without any significant difference at different temperatures.

**Figure 7:**
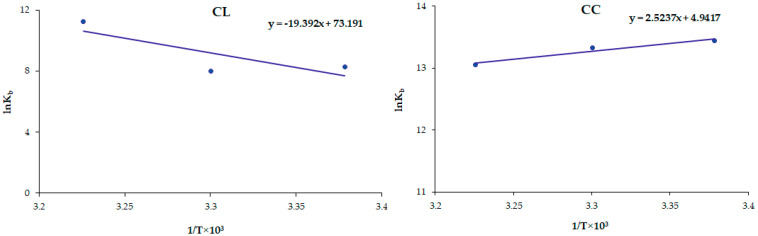
Van’t Hoff diagram of **CL** and **CC** interactions with HSA.

The most prominent interactions include non-covalent interactions, such as electrostatic forces, hydrogen bonds, van der Waals interactions, and hydrophobic forces, which are included in the binding process between drugs and biomolecules. The present contribution confirms the importance of the mentioned interactions by the sign and magnitude of the thermodynamic parameters (ΔS°, ΔH°, and ΔG°) (Figure 7).

The negative values of ΔG° indicate that the process is spontaneous in all cases (Table 3). The negative values obtained for ΔS° and ΔH° (Table 3) are associated with van der Waals forces and hydrogen bonds as the types of interactions between compound **CC** and HSA. On the other hand, for compound **CL**, positive values of both ΔS° and ΔH° are observed, and this indicates that hydrophobic force is the major binding force in the formed system HSA-**CL**. Also, in the case of the ligand, TΔS° > ΔH°, i.e., an entropy-driven interaction process was detected. This further confirms that hydrophobic forces play a key role in HSA’s interaction with **CL**.

#### 3.4.3. Active Site Confirmation and Molecular Docking Analysis

**Figure 8:**
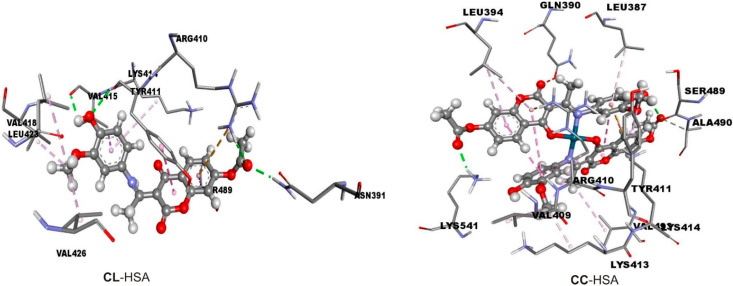
The hydrogen bonds (green dotted lines) and hydrophobic (rose pink dotted lines) docking interactions of the most stable conformations of the selected compounds with HSA.

In this study, the molecular interactions between the active binding sites of the HSA, AFP, and CEA receptors and the analyzed compounds (**CL** and **CC**) were investigated using molecular docking simulations. Prior to the binding process examination, the pockets and binding sites of the targeted receptors were identified. For this purpose, AMDock software 1.5.2 (University of Barcelona, Barcelona, Spain) was applied to configure and compute affinity maps for receptor molecules to be used with AutoDock4. The native bound ligand, ketoprofen, was first extracted from the protein structure, and a binding pocket analysis was performed. Following this, re-docking was conducted with the investigated compounds to generate the same docking pose as found in its co-crystallized form. Additionally, the molecular docking simulation was performed to examine the interaction of the tested compounds with AFP and CEA receptors. This step was needed to compare the binding affinities of the ligand, complex, and ketoprofen [60] and to correlate them with the experimental binding energy. The free energies of binding and most stable docking conformations of the investigated compounds are presented in Table 4 and Figure 8. It should be kept in mind that a lower inhibition constant (Ki) value and a more negative value of free energy of binding (ΔG_bind_) indicate better binding affinities.

The binding affinities of the compounds **CL** and **CC** toward the investigated receptors were ranked based on their lowest binding energies involved in complex formation at the active sites. The binding energies of the docked compounds to HSA were found to be in the range of −32.5 to −25.9 kJ mol^−1^, demonstrating significant binding potential towards HSA (Table 4). Based on the results presented in Table 4, it is clear that the Pd complex has lower binding energy values than its parent ligand. Notably, **CC** exhibited a lower binding energy than ketoprofen, highlighting its potential as a drug candidate. The results in Table 4 indicate very good agreement with the experimentally obtained results. Based on the results presented in Table 4, it is clear that the Pd complex has significantly lower values of binding energy than its parent ligand. Moreover, **CC** has the most pronounced activity against AFP and CEA receptors than to HSA. It is important to note that compound **CC** shows significant binding activity towards AFP, while its activity toward other receptors is somewhat weaker.

Molecular docking analyses revealed a variety of non-covalent interactions between the compounds and target receptors, primarily hydrogen bonds, π–alkyl interactions, π–π interactions, and π–cation interactions (Figure 8). Amino acids LYS, SER, and ARG at positions 541, 489, and 410, respectively, are crucial within the active sites of HSA, forming strong hydrogen bonds with **CC** (the lengths are 2.06, 2.12, and 2.15 Å). On the other hand, amino acids ASN, ARG, and VAL at positions 391, 410, and 415 form hydrogen bonds (the lengths are 2.04, 2.22, and 2.58 Å) with **CL**, playing a predominant role in the active site. Meanwhile, other amino acids contribute to weaker alkyl–π, π–π interactions, and π–cation interactions with the benzene rings and alkyl moieties of the investigated compounds (Figure 8). Figure S6 illustrates the interactions of **CL** and **CC** with AFP receptors. As shown in Figure S6, the most significant interactions include hydrogen bonds, alkyl–π, and π–π interactions. The amino acids that form hydrogen bonds with the ligands play a predominant role in the active sites of these receptors. These amino acids establish strong hydrogen bonds, whereas other amino acids create weaker alkyl–π and π–π interactions with the benzene ring and alkyl moiety of the investigated ligands.

## 4. Conclusions

This manuscript investigates the synthesis, structural characterization, and comparison of the biological activities of a coumarin–palladium(II) complex (**CC**) and coumarin ligand (**CL**). The structural properties of the ligand and its complex with Pd(II) were analyzed through spectroscopic techniques (NMR and IR) and theoretical methods (DFT), confirming a stable complexation through oxygen and nitrogen atoms. Biological studies revealed that both the ligand and the complex exhibit cytotoxic effects on several cancer cell lines, with the **CC** showing higher efficacy, particularly against HeLa cells. The complex induces oxidative stress, leading to caspase-dependent apoptosis, a mechanism likely related to reactive oxygen and nitrogen species (ROS/RNS) production. Additionally, spectrofluorimetric studies suggest significant binding interactions of both investigated compounds with human serum albumin (HSA), further indicating their potential as an anti-cancer agent. The manuscript concludes that the palladium complex enhances the biological activity of the coumarin ligand and could be a promising candidate for further anti-cancer research.

## Figures and Tables

**Table 1:** Experimental and theoretical (at B3LYP/6-311++G(d,p) level of theory) ^1^H and ^13^C NMR chemical shifts of **CC**.

^1^H			^13^C		
H Atom	Exp. [ppm]	Calc. [ppm]	C Atom	Exp. [ppm]	Calc. [ppm]
C4′–H	2.18	2.35	C2	168.7	163.3
C2′–H	2.31	2.14	C3	96.76	104.8
OCH_3_	3.75	3.64	C4	179.5	172.8
C5–H, C6–H, C8–H	7.00	6.97	C5	118.1	128.4
C2″–H, C5″–H, C6″–H	6.84	7.04	C6	127.1	117.7
R		0.998	C7	154.9	157.9
MAE [ppm]		0.14	C8	112.5	108.8
			C9	153.8	155.7
			C10	109.9	117.9
			C1′	175.7	174.4
			C2′	21.06	21.95
			C3′	161.4	174.7
			C4′	20.54	16.25
			C1″	128.6	142.2
			C2″	117.7	108.5
			C3″	147.7	146.9
			C4″	147.2	146.0
			C5″	112.8	113.3
			C6″	116.3	116.5
			OCH_3_	55.97	51.29
			R		0.989
			MAE [ppm]		5.14

**Table 2:** Stern–Volmer constants, binding constants, and number of binding sites of ligands and complexes with HSA at different temperatures.

Compound	Temperature (K)	K_SV_ (M^−1^)	k_q_ (M^−1^)	K_b_ (M^−1^)	n (HSA)
**CL**	296	9.56 × 10^4^	9.56 × 10^12^	3.86 × 10^3^	0.66
	303	7.69 × 10^4^	7.69 × 10^12^	2.98 × 10^3^	0.71
	310	7.27 × 10^4^	7.27 × 10^12^	7.64 × 10^4^	1.09
**CC**	296	2.95 × 10^5^	2.95 × 10^13^	6.87 × 10^5^	1.07
	303	2.72 × 10^5^	2.72 × 10^13^	6.14 × 10^5^	1.06
	310	2.80 × 10^5^	2.80 × 10^13^	4.67 × 10^5^	1.10

**Table 3:** The thermodynamic parameters of HSA-**CL** and HSA-**CC** systems at different temperatures.

Compound	Temperature (K)	ΔG° (kJ mol^−1^)	ΔS° (J mol^−1^K^−1^)	ΔH° (kJ mol^−1^)
**CL**	296	−18.89	608.51	161.23
	303	−23.15		
	310	−27.41		
**CC**	296	−33.14	−41.09	−20.98
	303	−33.43		
	310	−33.72		

**Table 4:** The calculated values of free energies of binding and inhibition constants of the investigated compounds with HAS, AFP, and CEA.

Conformations	ΔG_bind_ (kJ mol^−1^)	Ki (µM)
**Ketoprofen**-HSA	−26.0	27.4
**CL**-HSA	−25.9	29.0
**CC**-HSA	−32.5	1.94
**CL**-AFP	−37.7	0.25
**CC**-AFP	−38.5	0.18
**CL**-CEA	−34.5	0.92
**CC**-CEA	−37.9	0.23

## Data Availability

Data are contained within the article and Supplementary Materials.

## Supplementary Materials

The following supporting information can be downloaded at https://www.mdpi.com/article/10.3390/pharmaceutics16111437/s1, Figure S1. ^1^H NMR spectra of the coumarin complex **CC**; Figure S2. ^13^C NMR spectra of the coumarin complex **CC**; Table S1: IC_50_ values (μM) of treatment with **CL**, **CC,** and cisplatin, determined after 72 h using MTT and CV assays. Values from three independent experiments are expressed as the mean ± SD.; Figure S3: Cell viability. Compounds **CL**, **CC,** and cisplatin decreased the viability of cancer cells after 72 h of treatment in a dose-dependent manner. Human cancer cells Hela, A2780, MCF7, and HCT116 were treated with concentrations ranging from 0 to 200 μM for **CL** and **CC** and from 0 to 50 μM for cisplatin. Cell viability was determined using MTT (left) and CV (right) assays. Data are expressed as a percentage of the control (viability of untreated cells) ± SD of one representative from three independent experiments. *, *p* < 0.05 compared to the control; Figure S4: Effects of **CL** and **CC** on the viability of PECs. Compounds **CL** (left) and **CC** (right) showed reduced viability on mouse peritoneal exudate cells after 72 h compared to cancer cells. PECs were treated with concentrations ranging from 0 to 200 μM of both compounds. Viability was established using the CV assay. The data are presented as a percentage of the control ± SD triplicate culture. *, *p* < 0.05 compared to control; Figure S5: Treatment with **CL** and **CC** does not inhibit the proliferation of HeLa cells. HeLa cells (8 × 10^4^) were pre-stained with CFSE and treated with IC_50_ doses of **CL** and **CC** for 72 h. Data from three independent experiments are shown as the mean ± SD. n.s., not statistically significant in comparison to the control, one-way ANOVA with Bonferroni correction; Table S2: The optimized Cartesian coordinates and total energy for the ligand and complexes; Table S3: Some important calculated bond lengths [Å] and bond angles [°] of the investigated compounds; Figure S6: The hydrogen bonds (green dotted lines) and hydrophobic (rose pink dotted lines) docking interactions of the most stable conformations of the selected compounds with AFP.
