# Supplementary material for: A Comprehensive Evaluation of a Coumarin Derivative and Its Corresponding Palladium Complex as Potential Therapeutic Agents in the Treatment of Gynecological Cancers: Synthesis, Characterization, and Cytotoxicity
Source: Pharmaceutics. 2024 Nov 11;16(11):1437. doi: 10.3390/pharmaceutics16111437 (PMC11597210; doi:10.3390/pharmaceutics16111437)
Supplement: Supplementary file 1 [file pharmaceutics-16-01437-s001.zip › pharmaceutics-3289882-supplementary.pdf]

pharmaceutics

MDPI

Article

Supplementary Materials: A Comprehensive Evaluation of a Coumarin Derivative and Its Corresponding Palladium Complex as Potential Therapeutic Agents in the Treatment of Gynecological Cancers: Synthesis, Characterization, and Cytotoxicity

Mirela Jevtić, Marijana Stanojević Pirković, Teodora Komazec, Marija Mojić, Sanja Mijatović, Danijela Maksimović-Ivanić, Dušan Dimić, Zoran Marković, Dušica Simijonović, Dejan Milenković and Edina Avdović

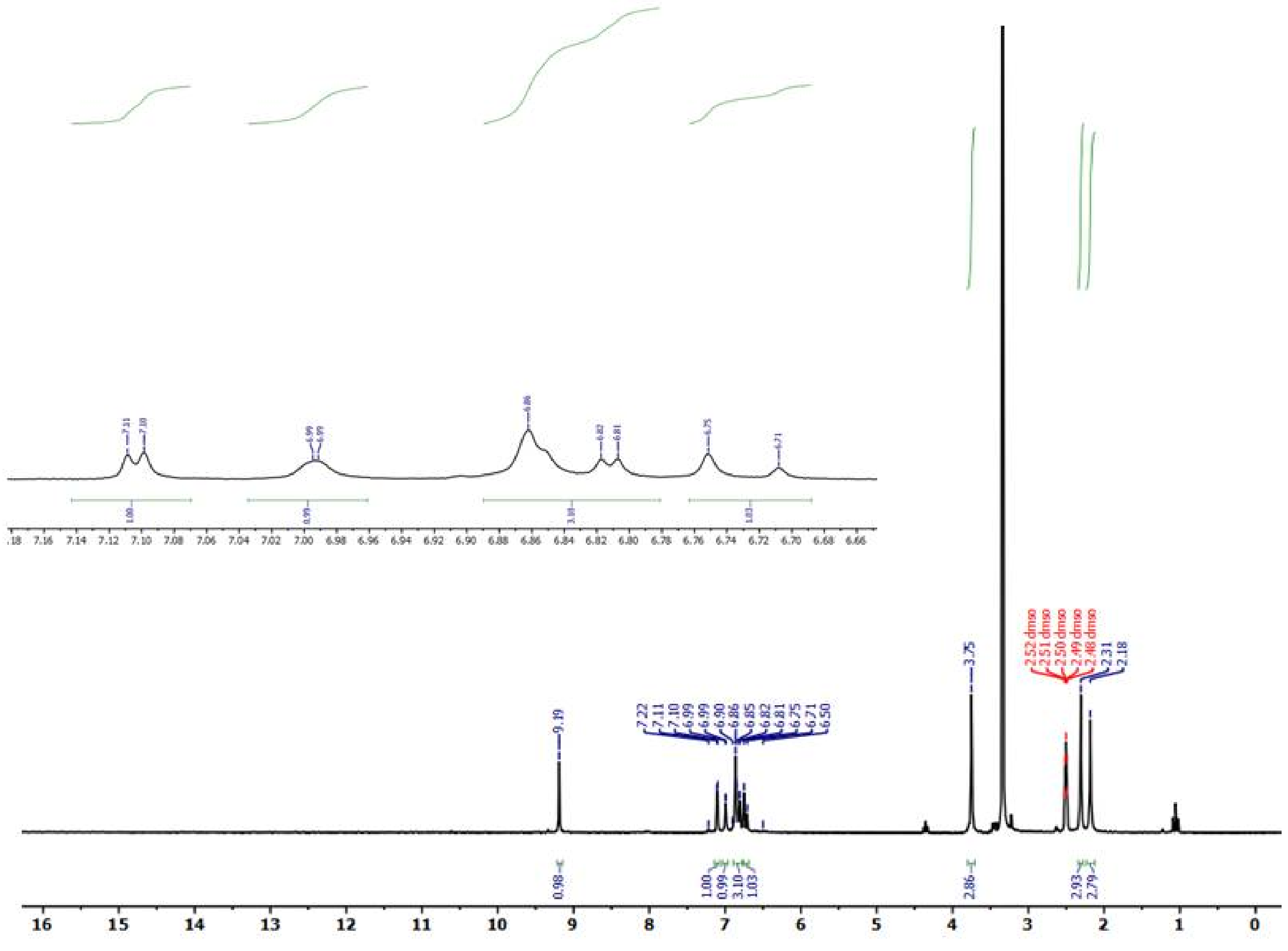

Figure S1. ^{1}H NMR spectra of the coumarin complex **CC**.

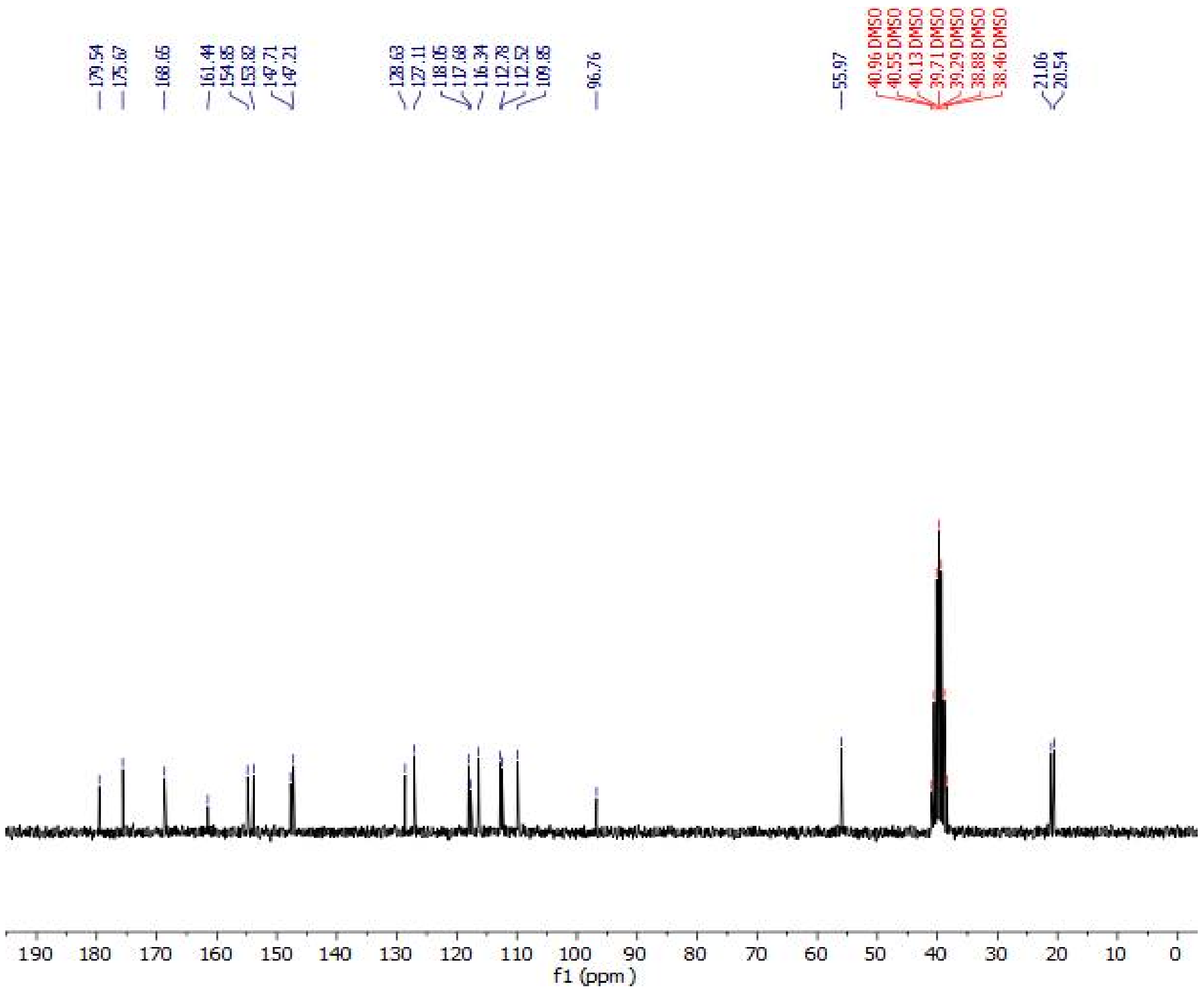

Figure S2. ^{13}C NMR spectra of the coumarin complex **CC.**

Table S1. IC_{50} values (μM) of treatment with **CL**, **CC** and cisplatin determined after 72 h by MTT and CV assays. Values from three independent experiments are expressed as mean ± SD.

Compound	HeLa		A2780		MCF7		HCT116		PEC
	IC_{50} [μM]								
	MTT	CV	MTT	CV	MTT	CV	MTT	CV	CV
**CL**	11.1±0.4	17.7±1.2	21.1±1.4	24.1±1.4	45.6±3.5	44.0±1.7	54.4±4.3	60.2±3.8	96.3
**CC**	17.9 ±1.5	19.7±0.1	40.7±0.5	42.0±4.0	45.1±3.7	39.1±2.3	47.2±1.8	50.8±1.1	92.9
**Cisplatin**	0.5±0.0	0.5±0.0	2.8±0.2	2.3±0.2	11.6±0.9	13.9±0.5	10.6±0.6	12.5±1.2	

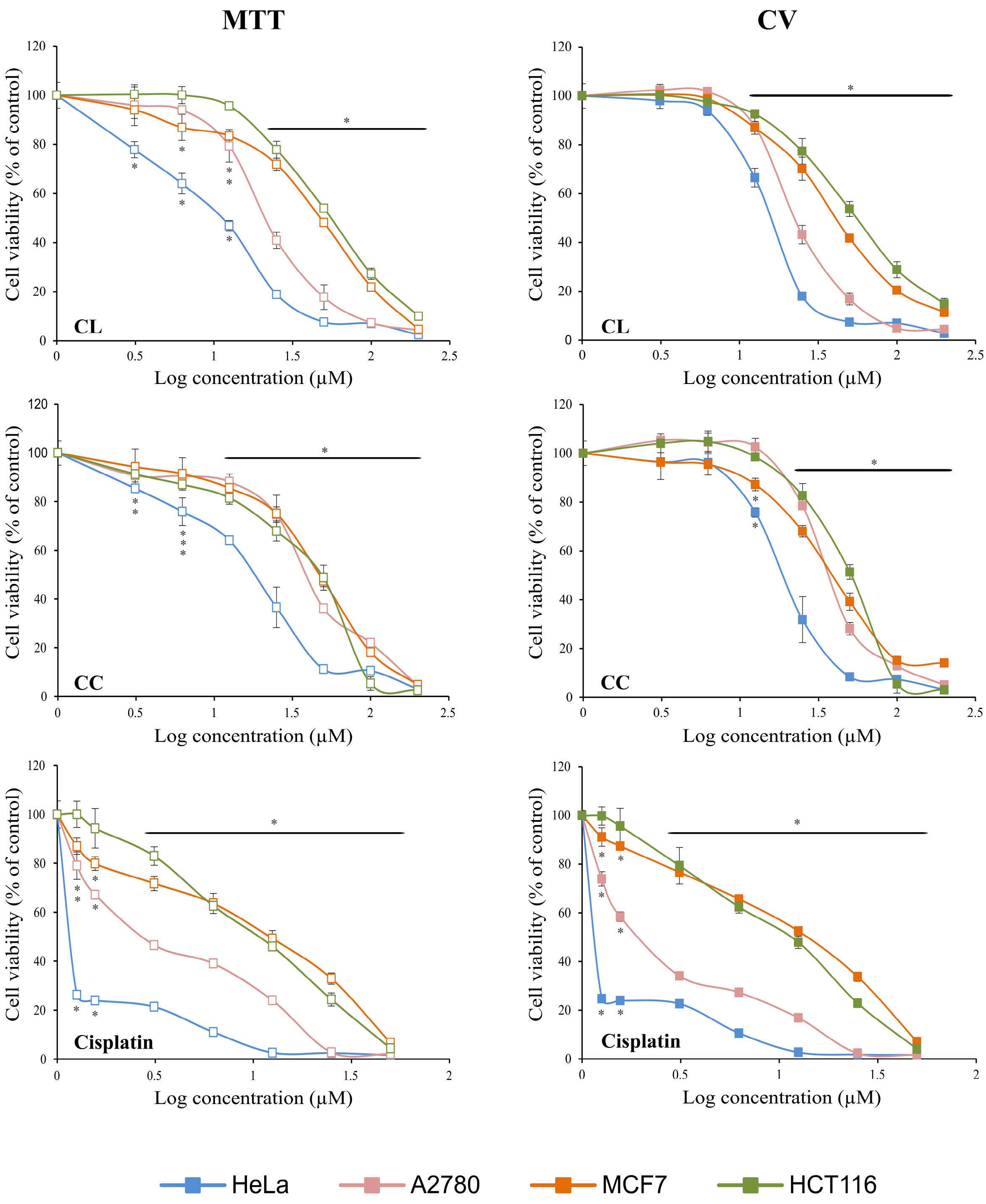

Figure S3. Cell viability. Compounds **CL**, **CC** and cisplatin decreased the viability of cancer cells after 72 h of treatment in a dose-dependent manner. Human cancer cells HeLa, A2780, MCF7 and HCT116 were treated with concentrations ranging from 0 to 200 µM for **CL** and **CC** and from 0 to 50 µM for cisplatin. Cell viability was determined using MTT (left) and CV (right) assays. Data are expressed as a percentage of control (viability of untreated cells) ± SD of one representative from three independent experiments. *, $p < 0.05$ compared to control.

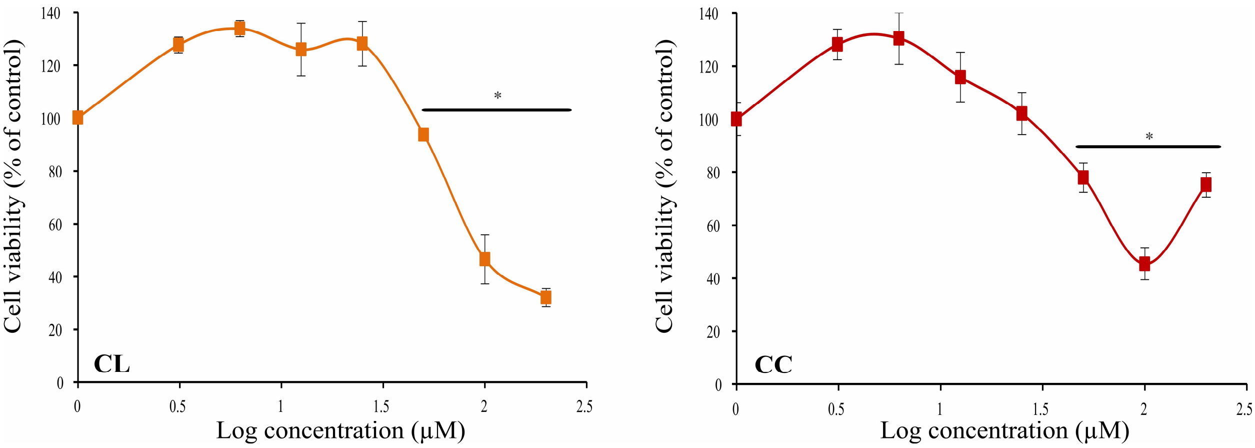

Figure S4. Effect of **CL** and **CC** on the viability of PEC. Compounds **CL** (left) and **CC** (right) showed less reduced viability on mouse peritoneal exudate cells after 72 h compared to cancer cells. PEC were treated with concentrations ranging from 0 to 200 μM of both compounds. Viability was established using the CV assay. The data is presented as a percentage of control ± SD triplicate culture. *, $p < 0.05$ compared to control.

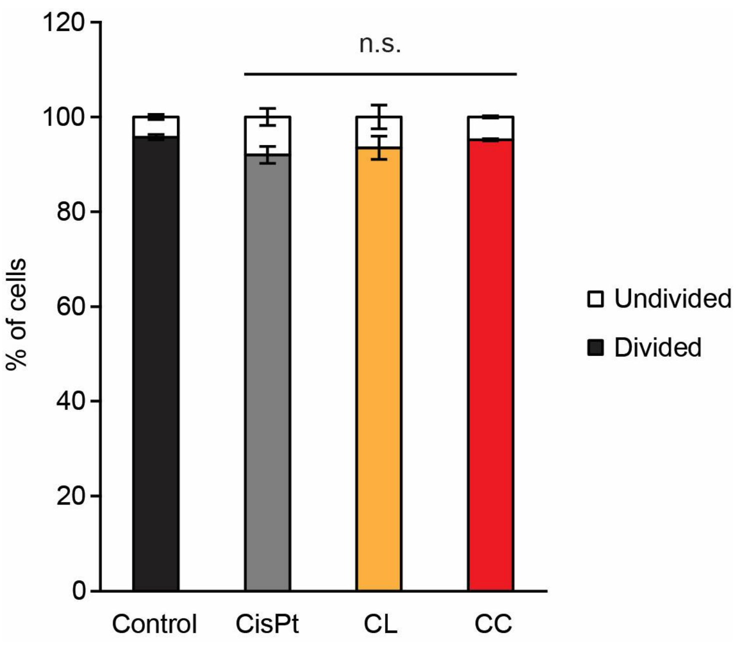

Figure S5. Treatment with **CL** and **CC** does not inhibit the proliferation of HeLa cells. HeLa cells (8 × 10^4) were pre-stained with CFSE and treated with IC_{50} doses of **CL** and **CC** for 72 hours. Data from three independent experiments are shown as the mean ± SD. n.s. not statistically significant in comparison to the control, one-way ANOVA with Bonferroni correction.

Table S2. The optimized Cartesian coordinates and total energy for the ligand and complexes.

	**CL**		
	B3LYP-D3BJ/6–311+G(d,p)		
	Energy = -1353.823247 a.u.		
	Charge = 0 Multiplicity = 1		
N	-1.99122500	-0.44255600	-0.16786100
C	-1.31531900	0.64963600	-0.49847100
C	0.09876900	0.66826400	-0.26794000
C	0.83530100	1.89258900	-0.51319800

C	0.78213500	-0.51044400	0.22380000
C	2.24175200	-0.42759600	0.36980300
C	2.89858800	0.76279000	0.06426400
C	-3.39591000	-0.66656500	-0.24628900
C	-3.85869900	-1.79015600	-0.92164500
C	-4.28727600	0.19103800	0.41000900
C	-5.22758900	-2.05562600	-0.96615200
H	-3.15785200	-2.44840700	-1.41879400
C	-5.64877200	-0.07185100	0.35366900
H	-3.90910300	1.03867700	0.96230200
C	-6.12320600	-1.20271700	-0.33972400
O	0.20019100	-1.58154400	0.51148700
C	-2.05558000	1.77927200	-1.14494800
H	-3.03502100	1.44823200	-1.48153900
H	-2.18041800	2.60896300	-0.44633500
H	-1.48108100	2.16530400	-1.98346600
O	2.21926900	1.86758200	-0.35231800
O	0.39200900	2.97560100	-0.84022900
C	3.00018100	-1.52441400	0.80416500
C	4.37526700	-1.43132300	0.92657000
C	4.99556900	-0.22193900	0.60825700
C	4.28248600	0.88213900	0.17866800
H	4.77768300	1.81352700	-0.06022000
H	4.97146900	-2.26967000	1.26180400
H	2.48307900	-2.44486400	1.04027900
O	-6.62797100	0.67611400	0.93445200
O	6.37330700	-0.09727500	0.78789700
C	7.19374500	-0.46494800	-0.24579200
O	6.77153200	-0.87465600	-1.29705900
C	8.63070100	-0.27886800	0.13394500
H	9.27037800	-0.56528600	-0.69724300
H	8.80861300	0.76469100	0.40147100
H	8.86051600	-0.88857700	1.01022200
H	-5.60683500	-2.92238100	-1.49276800
C	-6.25092000	1.85908900	1.64693400
H	-7.17902100	2.29891300	2.00306200
H	-5.60980100	1.61026600	2.49624300
H	-5.73819300	2.56077900	0.98421300
O	-7.46037700	-1.45793900	-0.38752100
H	-7.91743000	-0.75854700	0.10253400
H	-1.38942200	-1.20701000	0.18404200
**CC-*cis***			
B3LYP-D3BJ/6–311+G(d,p)/def2-TZVPD			
Energy = -2834.492748 a.u.			
Charge = 0 Multiplicity = 1			
N	-1.94819800	0.70853100	-0.95664600
C	-2.02285800	2.01191300	-0.96259400
C	-0.95254900	2.83513900	-0.41393300
C	-1.31341500	4.11867600	0.14682000
C	0.40016400	2.45448600	-0.53280400
C	1.41467700	3.47498800	-0.29579700

C	1.01469800	4.73708900	0.14957000
C	-2.93667000	-0.12398800	-1.56602200
C	-2.52072800	-1.15745400	-2.40207300
C	-4.29512300	0.02109800	-1.26042700
C	-3.45154600	-2.03667700	-2.94818600
H	-1.46693000	-1.27629600	-2.61870400
C	-5.21980500	-0.85170100	-1.80985700
H	-4.60899400	0.77149500	-0.55244500
C	-4.80044100	-1.89202800	-2.65735500
O	0.80604900	1.29089200	-0.87034400
N	-1.31512000	-1.78098300	0.72731500
C	-0.78546300	-2.95046500	0.99044600
C	0.53102100	-3.33400300	0.50417600
C	0.78792200	-4.75464900	0.32094200
C	1.56060500	-2.39403500	0.30177800
C	2.91780200	-2.88916000	0.09834700
C	3.13206900	-4.26607100	0.01969700
C	-2.59826100	-1.41788000	1.24536800
C	-3.73024200	-2.18311600	0.98624300
C	-2.71336200	-0.21498300	1.95392500
C	-4.97387400	-1.77732900	1.46539300
H	-3.65181700	-3.07560800	0.38168100
C	-3.95581700	0.20206500	2.40480700
H	-1.83034800	0.38586900	2.11855800
C	-5.09402400	-0.59191900	2.17194300
H	-5.86406200	-2.35837500	1.26052300
O	1.42010600	-1.12375800	0.32489300
Pd	-0.31596000	-0.26827700	-0.20784700
C	4.41140300	-4.78676900	-0.16508500
C	5.47191100	-3.90509500	-0.27315900
C	5.29193800	-2.51954200	-0.21103400
C	4.01519300	-2.02355000	-0.02749200
H	3.83152400	-0.95887000	0.02413300
H	6.14077100	-1.85677500	-0.29786500
H	4.56316400	-5.85538800	-0.22952100
C	-1.47943800	-3.91124300	1.93187000
H	-2.08426600	-3.35850400	2.64878300
H	-2.12249000	-4.60502500	1.39141800
H	-0.74002800	-4.51272000	2.45521000
C	-3.15033700	2.73561600	-1.66395200
H	-3.51688600	2.13896500	-2.49802700
H	-3.97793900	2.93528100	-0.98298300
H	-2.80008300	3.70031800	-2.02428300
O	-0.29044500	5.03037500	0.39821000
O	-2.43027400	4.47473600	0.45694700
O	2.10818800	-5.15354200	0.09880700
O	-0.03260900	-5.64054000	0.31988300
C	2.78184600	3.22589500	-0.48974700
C	3.72115900	4.21391000	-0.26349300
C	3.28473700	5.46980600	0.17028500
C	1.94591000	5.74587300	0.38526000

H	1.62469100	6.71727900	0.73507700
H	4.77386700	4.02978600	-0.42265200
H	3.07619500	2.23945000	-0.82212000
O	-4.21273400	1.36051800	3.08092400
O	6.71960400	-4.46325600	-0.53236600
C	7.78828200	-4.16056400	0.27859400
C	9.01796100	-4.86307800	-0.22341800
H	9.86226200	-4.61626800	0.41604300
H	8.85202900	-5.94253200	-0.22567300
H	9.22325400	-4.56156500	-1.25281900
O	7.71360500	-3.44630000	1.23837500
H	-3.14144700	-2.84275600	-3.60086600
O	-6.56775700	-0.83214900	-1.57868700
O	4.18631500	6.47954600	0.48874400
C	5.09842400	6.90373300	-0.45066300
O	5.15226800	6.46990500	-1.56650000
C	5.97395300	7.97132900	0.14143800
H	6.69399600	8.30282200	-0.60305900
H	5.36042600	8.81256600	0.47099000
H	6.49234600	7.58053200	1.01969100
C	-3.16773600	2.33413800	3.18011800
H	-3.59860500	3.17760000	3.71427800
H	-2.32087000	1.93594400	3.74603200
H	-2.84670800	2.66123000	2.18874100
O	-6.31633600	-0.16537100	2.60123500
H	-6.18252800	0.66260000	3.08446700
C	-7.11797800	0.26747500	-0.85267700
H	-8.19747600	0.13639600	-0.88947200
H	-6.78843600	0.25785200	0.18774200
H	-6.84620200	1.21494900	-1.32820600
O	-5.71074700	-2.75750800	-3.17640100
H	-6.58493500	-2.48834300	-2.86167100

CC-*trans*

B3LYP-D3BJ/6–311+G(d,p)/def2-TZVPD

Energy = -2834.494896 a.u.

Charge = 0 Multiplicity = 1

N	-0.16443700	-1.95942300	0.36151200
C	0.71218500	-2.88480000	0.04409600
C	2.04943300	-2.57638600	-0.42733500
C	2.82273700	-3.64395300	-1.06122000
C	2.63590100	-1.29660600	-0.30022900
C	4.07401000	-1.16445100	-0.50717100
C	4.80429200	-2.27032000	-0.93848200
C	-1.46875800	-2.35033400	0.81188000
C	-1.76979800	-2.40189000	2.16570400
C	-2.45001300	-2.63256000	-0.14187200
C	-3.04676400	-2.78352300	2.58088600
H	-1.00794700	-2.15148100	2.89279100
C	-3.72296400	-2.99361600	0.27277700
H	-2.20500000	-2.54331700	-1.19090400
C	-4.02232400	-3.08648100	1.64325800

O	2.03732500	-0.21733600	0.02142000
N	0.28692900	2.06616000	-0.19247000
C	-0.65008700	2.84630500	-0.66910000
C	-1.99571400	2.36675000	-0.94080800
C	-2.80966000	3.10968800	-1.89260700
C	-2.54365300	1.25327200	-0.27186300
C	-3.99123500	1.07506100	-0.30407600
C	-4.74754400	1.85692300	-1.17759300
C	1.59698500	2.58120800	0.06375800
C	2.41889800	2.98344700	-0.98230800
C	2.07202800	2.59744600	1.37824200
C	3.71090600	3.43719800	-0.72127600
H	2.05664000	2.92934000	-2.00048600
C	3.36251400	3.03898200	1.63422600
H	1.42871900	2.24600600	2.17240900
C	4.19020000	3.46576600	0.57921700
H	4.36761300	3.74431000	-1.52531300
O	-1.89293300	0.38711400	0.40350800
Pd	0.06985600	0.05648100	0.15062400
C	-6.12919000	1.69499900	-1.27195900
C	-6.73780600	0.75456300	-0.45931700
C	-6.01256300	-0.02531500	0.44597600
C	-4.64250600	0.14083600	0.51419100
H	-4.04607000	-0.43847400	1.20248600
H	-6.51853700	-0.73418600	1.08606600
H	-6.70505800	2.29522200	-1.96288500
C	-0.37738600	4.32433500	-0.84090400
H	0.38183700	4.64609300	-0.13033800
H	-0.02315900	4.54037200	-1.84935400
H	-1.28997800	4.89764000	-0.69984900
C	0.33517500	-4.33599300	0.24769600
H	-0.43104100	-4.42279300	1.01315300
H	-0.05049400	-4.75800200	-0.68219700
H	1.20352800	-4.92910100	0.51973500
O	4.19647500	-3.44768500	-1.22864000
O	2.40170000	-4.69355400	-1.48118700
O	-4.16960000	2.78566000	-1.98014900
O	-2.43059200	3.96232600	-2.65789900
C	4.74759700	0.04076200	-0.26057000
C	6.11520400	0.13671800	-0.42756600
C	6.81867600	-0.99603500	-0.84860500
C	6.18532800	-2.19824700	-1.11194500
H	6.74098200	-3.06139600	-1.45155600
H	6.63337400	1.06468500	-0.23038500
H	4.16967400	0.89117400	0.06427700
O	3.95785700	3.08942200	2.86477400
O	-8.10738500	0.56628700	-0.62617400
C	-8.94850800	0.79111700	0.44044500
O	-8.57010700	1.17045000	1.51212200
C	-10.36664500	0.49974700	0.03893300
H	-11.02488200	0.66872200	0.88792100

H	-10.65508600	1.14552000	-0.79329500
H	-10.45111200	-0.53367500	-0.30392100
O	-4.78377700	-3.25718500	-0.55063000
O	8.18618000	-0.94126100	-1.09986500
C	9.04514100	-0.55641000	-0.09735200
O	8.69047100	-0.29706000	1.01812500
C	10.45313200	-0.52695600	-0.62087600
H	11.13147900	-0.24256300	0.18013600
H	10.72428400	-1.50925100	-1.01327200
H	10.52507100	0.18604100	-1.44521000
H	-3.30131900	-2.83337100	3.63210200
C	3.25350000	2.54087300	3.97488100
H	3.92186900	2.63157900	4.82837200
H	2.33335200	3.10039100	4.16879000
H	3.01644000	1.48711500	3.80073200
O	5.46684800	3.86699700	0.83001400
H	5.62213900	3.78274000	1.78100000
C	-4.69513600	-2.82871300	-1.90862500
H	-5.67483000	-3.01043100	-2.34534100
H	-4.45975400	-1.76176700	-1.95637600
H	-3.94163900	-3.40459700	-2.45356300
O	-5.28137900	-3.42253700	2.04135500
H	-5.80407800	-3.58138100	1.24304800

Table S3. Some important calculated bond lengths [Å] and bond angles [°] of investigated compounds.

Bond length	**Cl**	**CC-*cis***	**CC-*trans***
C2`–N	1.332	1.306	1.314
C2`–C3	1.423	1.457	1.451
C3–C4	1.452	1.410	1.413
C4–O	1.250	1.278	1.275
(C2`–)N–Pd		2.044	2.040
(C2``–)N–Pd		2.040	2.050
(C4–)O–Pd		2.032	1.991
(C4`–)O–Pd		2.007	2.006
**Bond angle**			
N–C2`–C3	118.3	121.3	122.9
C2`–C3–C4	120.4	121.3	123.1
C3–C4–O	123.6	124.9	126.4
(C2`–)N–Pd–N(–C2``)		97.54	176.3
(C4–)O–Pd–O(–C4`)		86.33	176.1
(C4–)O–Pd–N(–C2`)		87.41	89.10
(C4–)O–Pd–N(–C2``)		171.5	91.11
(C4`–)O–Pd–N(–C2`)		172.5	92.15
(C4`–)O–Pd–N(–C2``)		89.19	87.89
τ_4*		0.113	0.054

*The τ_4 parameter is calculated according paper Yang et al., 2007 [58].

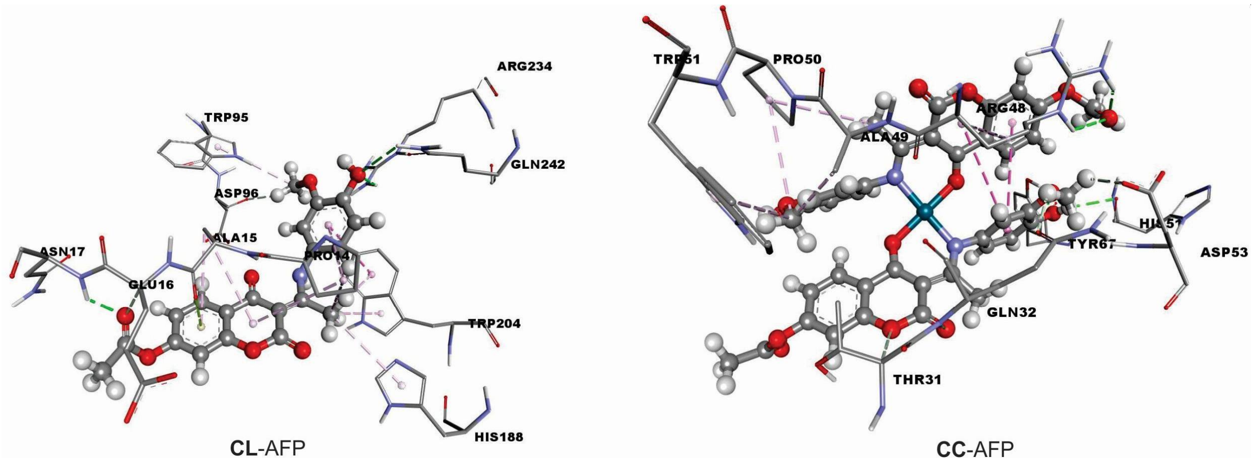

Figure S6. The hydrogen bond (green dotted lines) and hydrophobic (rose pink dotted lines) docking interactions of the most stable conformations of selected compounds with AFP.
